# *In vivo* evaluation of binder jet 3D-Printed monetite, brushite, and octacalcium phosphate: A comparative study for bone regeneration in a rat calvarial defect model

**DOI:** 10.1371/journal.pone.0349259

**Published:** 2026-05-15

**Authors:** Ticomporn Luangwattanawilai, Faungchat Thammarakcharoen, Autcharaporn Srion, Kasem Rattanapinyopituk, Jintamai Suwanprateeb, Ruedee Hemstapat

**Affiliations:** 1 Department of Pharmacology, Faculty of Science, Mahidol University, Bangkok, Thailand; 2 Biofunctional Materials and Devices Research Group, National Metal and Materials Technology Center (MTEC), Pathum Thani, Thailand; 3 Department of Pathology, Faculty of Veterinary Science, Chulalongkorn University, Bangkok, Thailand; 4 Thammasat University Center of Excellence in Computational Mechanics and Medical Engineering, Thammasat University, Pathum Thani, Thailand; Universidade de Trás-os-Montes e Alto Douro: Universidade de Tras-os-Montes e Alto Douro, PORTUGAL

## Abstract

**Background:**

Three-dimensional (3D)-printed hydroxyapatite (3DP-HA), fabricated via binder jetting of calcium sulfate-based powders followed by phase conversion, has demonstrated bone regeneration efficacy in both *in vitro* and *in vivo* studies. However, the inherently low solubility nature of hydroxyapatite (HA) led to slow resorption, which may impede new bone formation. This study aimed to evaluate the *in vivo* bone regeneration efficacy of three newly developed resorbable 3D-printed calcium phosphate scaffolds, including brushite (3DP-BRU), monetite (3DP-MO), and octacalcium phosphate (3DP-OCP), fabricated using the similar technology as 3DP-HA.

**Methods:**

The scaffolds were implanted in a rat calvarial defect model and compared with control groups, including 3DP-HA and two commercial bone grafts: bovine bone graft (BBG) and freeze-dried bone allograft (FDBA). Bone regeneration and material resorption were assessed using micro-computed tomography (micro-CT), histological, and immunohistochemical analyses.

**Results:**

Micro-CT and histological evaluations demonstrated that 3DP-MO and 3DP-BRU scaffolds significantly enhanced new bone formation and bone cell activities within the defect sites compared with the controls. Furthermore, both 3DP-MO and 3DP-BRU exhibited considerably lower residual graft material compared to the controls, indicating superior resorption characteristics.

**Conclusion:**

Resorbable 3D-printed calcium phosphate scaffolds, particularly 3DP-MO and 3DP-BRU, exhibit superior resorbability and enhanced bone regeneration compared with conventional materials. These findings highlight their potential as promising biomaterials for clinical application in bone defect repair.

## Introduction

Autologous bone grafting remains the gold standard for repairing bone defects due to its superior osteogenic, osteoconductive, and osteoinductive properties. However, its clinical application is restricted by factors such as donor site morbidity, insufficient graft availability, and the requirement for an additional surgical procedure [1,2]. Alternative materials such as allografts and xenografts also exhibit several drawbacks, including risks of immune rejection, potential disease transmission [3,4], and diminished osteogenic and osteoconductive performance resulting from processing treatment [5]. Therefore, the development of a safe, effective, and readily available bone graft alternative is still needed.

Hydroxyapatite (HA), a major inorganic component of natural bone, has been extensively utilized in bone grafting owing to its excellent biocompatibility and osteoconductive properties. Nonetheless, conventional HA scaffolds produced by high-temperature sintering typically exhibit high-crystallinity and large crystal size, leading to slow *in vivo* resorption and limited bone remodeling capacity [6]. Achieving an optimal balance between new bone formation and scaffold resorption is essential for maintaining volumetric stability and mechanical integrity of bone grafts [7]. Recently, other calcium phosphate phases with higher solubility than HA, such as brushite (BRU), monetite (MO), and octacalcium phosphate (OCP), have attracted increasing attention as potential alternatives [8–10]. Brushite, first identified by Mirtchi and Lemaître in 1989 [11], has since been utilized for bone regeneration in various forms, including injectable pastes, blocks, and granules [12–15]. Several studies have reported that brushite exhibits a faster resorption rate than HA [16,17]. Monetite, the anhydrous form of brushite, has shown the ability to achieve a favorable balance between resorption and new bone formation [18,19], with osteogenic potential comparable to autologous bone grafts [19]. OCP, regarded as a precursor to biological apatite in bones and teeth [20], has also been explored as a bone substitute material and found to promote bone regeneration effectively [21–23]. Moreover, OCP exhibits superior osteoconductivity and biodegradability compared with HA and β-tricalcium phosphate (β -TCP) [24].

Recent advancements in 3D printing technology have introduced new opportunities for applications in bone grafting and tissue engineering. The integration of 3D printing into scaffold fabrication offers significant advantages, enabling the production of bioceramic grafts with precisely tailored geometries and customizable physicochemical properties [25]. Our group has previously developed 3D-printed hydroxyapatite (3DP-HA) using binder jet 3D printing combined with a low-temperature phase transformation process, resulting in materials with reduced crystallinity, improved osteoconductivity, and enhanced resorbability [26].

Although binder jetting typically yields parts with lower mechanical strength compared to other 3D printing methods, mainly due to high porosity and weak interparticle bonding, its major advantage lies in the fast-printing ability to fabricate complex structures at low temperatures via layer-by-layer deposition of a liquid binder onto a powder bed, without the need for polymer carriers or high-temperature post-processing [27]. Clinically, 3DP-HA has been demonstrated to be safe and effective for alveolar ridge preservation [28] and for the fabrication of personalized bone block grafts precisely matched to patient-specific anatomical defects [29]. Furthermore, the dual functionality of 3DP-HA, combining drug delivery capability with bone regenerative performance, has been validated in both preclinical and clinical studies [30,31].

Despite these promising results, 3DP-HA remains only partially resorbable due to its inherently low solubility of the hydroxyapatite phase [32]. To address the resorption limitation of 3DP-HA, this low-temperature fabrication technique was modified to successfully produce various monophasic calcium phosphate phases with greater solubility, including brushite (3DP-BRU) [33], monetite (3DP-MO) [34], and octacalcium phosphate (3DP-OCP) [35]. Although these calcium phosphate phases have individually demonstrated promising osteogenic and resorption characteristics, previous studies have largely evaluated them under different fabrication conditions, scaffold architectures, and porosity profiles, making direct biological comparisons difficult. Importantly, a systematic head-to-head *in vivo* comparison of binder jet–printed monophasic BRU, MO, and OCP scaffolds fabricated under identical architectural and porosity conditions has not yet been reported. Consequently, the specific influence of calcium phosphate phase composition independent of structural design parameters on bone regeneration and scaffold resorption remains unclear.

Based on the differences in physicochemical properties among calcium phosphate phases, we hypothesized that 3D-printed calcium phosphate scaffolds with higher phase solubility would exhibit accelerated material resorption and promote more effective coupled bone remodeling compared with conventional 3DP-HA. Furthermore, it was hypothesized that controlled resorption of these more soluble phases would facilitate enhanced new bone formation while maintaining structural stability within the defect site. This study aimed to evaluate and compare the *in vivo* bone regenerative performance of the developed scaffolds, with particular emphasis on comparing the newly developed, more soluble 3D-printed calcium phosphate (3DP-CaP) scaffolds with the original 3DP-HA and commercial bone grafts in a rat calvarial defect model. The investigation focused on assessing their biocompatibility, bone formation capacity, and scaffold resorption behavior. The ultimate goal was to identify the most promising calcium phosphate phase for the development of next-generation 3D-printed bone grafts that achieve an optimal balance between controlled resorption and enhanced osteogenesis, thereby improving clinical outcomes in bone regeneration.

## Materials and methods

### Sample Preparation

Calcium sulfate–based powder (VisiJet PXL Core, 3D Systems, Rock Hill, SC, USA) was used as the raw material and loaded into a binder jet 3D printer (ProJet 160, 3D Systems, Rock Hill, SC, USA) to fabricate spherical granules with a diameter of 1.3 mm according to the designed Stereolithography (STL) file. Printing was performed using an aqueous binder (VisiJet PXL Clear, 3D Systems, Rock Hill, SC, USA), which bonded the powder particles and solidified by hydration reaction, thereby eliminating the need for thermal curing or post-processing. For the fabrication of 3DP-MO, the as-printed granules were immersed in 2 M disodium hydrogen phosphate solution (Sigma-Aldrich, St. Louis, MO, USA) adjusted to pH 5.0 and maintained at 100 °C for 48 h. For 3DP-BRU, the as-printed granules were immersed in 1.5 M disodium hydrogen phosphate solution (Sigma-Aldrich, St. Louis, MO, USA) at pH 6.5 and 37 °C for 48 h. For 3DP-OCP, the transformation was carried out in two stages: first, the 3D-printed calcium sulfate samples were converted into brushite under the same conditions as for 3DP-BRU; subsequently, the brushite-converted samples were immersed in 1.5 M disodium hydrogen phosphate solution adjusted to pH 8.0 and heated at 65 °C for 48 h to induce transformation into OCP. For 3DP-HA, used as a comparative sample, the 3D-printed calcium sulfate granules were converted by immersion in 1.0 M disodium hydrogen phosphate solution (Sigma-Aldrich, St. Louis, MO, USA) at 100 °C for 48 h. After phase transformation, all samples were thoroughly rinsed and cleaned with de-ionized water and oven-dried. To ensure structural and chemical consistency across the experimental groups, all 3D-printed samples were fabricated in a single batch using a single lot of raw calcium sulfate powder and binder liquid. Following the build, all samples for each specific material group (3DP-HA, 3DP-MO, 3DP-BRU, and 3DP-OCP) were subjected to their respective phase conversion, washing, and drying protocols simultaneously in a single batch. This consolidated approach was designed to eliminate inter-batch variability in phase transformation kinetics and porosity development.

The physicochemical characteristics of the resulting 3DP scaffolds, including phase purity (XRD/FTIR), porosity, and compressive strength, were previously determined and summarized in **Table 1** [26,33–35]. The compressive strength of the scaffolds (1.55–7.65 MPa) was within the range reported for human cancellous bone (0.64–14.44 MPa [36], ensuring that the materials remain intact during surgical handling. The resulting materials were sterilized by gamma irradiation at a dose of 25 kGy (2.5 Mrad) prior to *in vivo* evaluation.

**Table 1:** Physicochemical and structural properties of the 3D-printed scaffolds.

Samples	Phase Composition (XRD/FTIR)	Porosity (%)	Compressive Strength (MPa)	References
3DP-HA	Hydroxyapatite	65.90	7.32 ± 0.54	[26,35]
3DP-MO	Monetite	70.63	1.55 ± 0.32	[34]
3DP-BRU	Brushite	42.20	3.95 ± 0.34	[33]
3DP-OCP	Octacalcium Phosphate	53.52	7.65 ± 0.46	[35]

**Fig 1:**
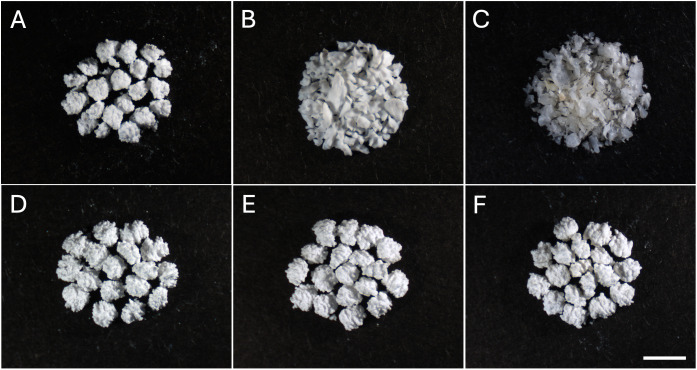
Images of 3D-printed calcium phosphate scaffolds and commercial bone substitute materials. (A) 3DP-HA; **(B)** BBG; **(C)** FDBA; (D) 3DP-MO; (E) 3DP-BRU (F) 3DP-OCP. Scale bar = 2 mm.

For comparative purposes, two clinically established bone graft materials were employed as reference controls: a human-derived freeze-dried bone allograft (FDBA, granule < 2.0 mm; Maxgraft, Botiss Biomaterials, Germany) and a bovine-derived bone xenograft (BBG; Bio-Oss, granule 1.0–2.0 mm, Geistlich Pharma, Switzerland). These materials were selected as representative allograft and xenograft grafting strategies with well-documented clinical use and distinct resorption characteristics, and are commonly employed as benchmark comparators in bone regeneration studies [37,38]. The representative images of the 3D-printed samples and commercial bone grafts are shown in Fig 1.

### Animals and Experimental design

The experimental protocol was designed, conducted, and reported in accordance with the ARRIVE guidelines [39] and was approved by the Institutional Animal Care and Use Committee (IACUC) of the Faculty of Science, Mahidol University, Thailand (Approval No: MUSC 64-022-571). Adult male Wistar rats (7–8-weeks old; 250–270 g) were obtained from Nomura Siam International Co., Ltd. (Bangkok, Thailand). Animals were housed in pairs under standard laboratory conditions (12-hour light/dark cycle; temperature 22 ± 1˚C; relative humidity 30–70%) with *ad libitum* access to standard rat chow and water. Animals were acclimatized for 5–7 days prior to study initiation in a facility accredited by the Association for the Assessment and Accreditation of Laboratory Animal Care (AAALAC). All surgical and implantation procedures were performed when the animals were 8–9 weeks of age.

Fifty-four male Wistar (8–9 weeks old; 300–320 g) rats were randomly assigned to two main groups according to the designated euthanasia timepoints: 4 weeks (n = 27) or 12 weeks (n = 27) post-implantation. Animals were further allocated into six experimental groups based on the implanted materials. Material allocation was performed prior to surgery using simple randomization by random drawing. Each animal was assigned a unique identification number, and biomaterials were randomly allocated to individual defects. To standardize surgical handling and reduce procedural variability, the left calvarial defect was designated as the control site. For each animal, the control material (3DP-HA, BBG, or FDBA) was randomly assigned, and the experimental scaffold implanted in the contralateral defect was independently randomized. Allocation of materials was determined before implantation to avoid operator bias. Six implant materials (3DP-HA, 3DP-MO, 3DP-BRU, 3DP-OCP, BBG, and FDBA) were distributed accordingly across defects. Each calvarial defect was defined a priori as an independent experimental unit for histomorphometric evaluation because defects received different biomaterials, were spatially separated, and were analyzed independently. Based on this design, nine defects were included per material group at each time point. The bilateral implantation design allowed each animal to contribute two independent defects while reducing inter-animal variability and minimizing animal use in accordance with the 3Rs principle.

**Fig 2:**
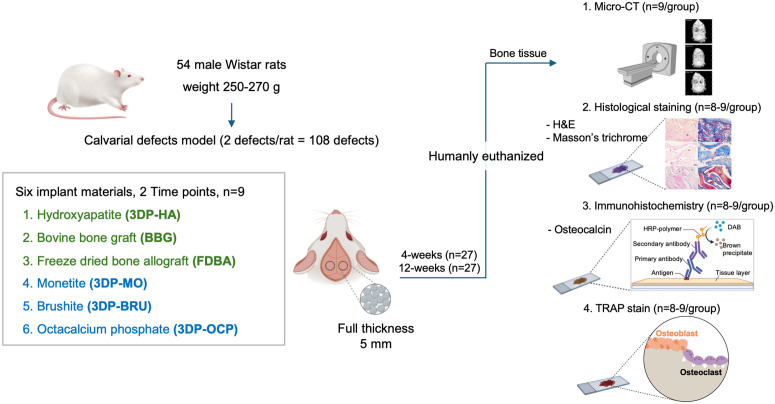
Schematic diagram summarizing the experimental design (Created with BioRender.com/Mahidol University).

After implantation, the general health of the animals was monitored weekly throughout the study period. At each study endpoint, the animals were humanely euthanized via intraperitoneal injection of an overdose of thiopental (150 mg/kg; Anesthal^®^; Scott-Edil Pharmacia Ltd., Solan, India) combined with xylazine (10 mg/kg; L.B.S. Laboratory Ltd. Part., Bangkok, Thailand). Calvarial bone tissues were then collected for further analyses, including bone histomorphometry/micro-CT, histological, and immunohistochemical examinations (**Fig 2**).

### Implantation

To prevent postoperative infection, animals were administered cefazolin sodium (20 mg/kg; Cefaben^®^, L.B.S. Laboratory Ltd., Bangkok, Thailand) via subcutaneous injection 30 minutes before surgery and once daily for four consecutive days thereafter. Under general anesthesia induced by inhalation of 3–5% of isoflurane (Attane™, Piramal Critical Care, Inc., USA) via an endotracheal tube, bilateral full-thickness calvarial defects (5 millimeters in diameter) were created in the center of each parietal bone using a trephine drill (Saeshin Precision Co., Ltd.; Daegu, Korea). Samples weighing 0.01 g were implanted into each defect site. Suturing of the pericranium over the samples was performed using a 4–0 non-absorbable silk suture (Ethicon^®^, Inc., Livingston, United Kingdom) to secure the samples in place, followed by skin closure using the same suture material. Postoperatively, tramadol (10 mg/kg; Tramada-100^®^, L.B.S. Laboratory Ltd., Bangkok, Thailand) was administered once daily for three consecutive days to relieve pain.

### Histological procedures and analysis

Following euthanasia, the calvarial bones containing the samples were harvested and fixed in 10% neutral buffered formalin for 48 hours, then preserved in 0.02% sodium azide (Cat#S2002, Sigma-Aldrich, MO, USA) prepared in freshly made phosphate-buffered saline (PBS; NaCl, KCl, Na_2_HPO_4_, and KH_2_PO_4_) at 4 °C until micro-CT analysis. After micro-CT analysis, the specimens were decalcified in 10% ethylenediaminetetraacetic acid (EDTA, Elago Enterprises Pty Ltd., New South Wales, Australia) at room temperature for four weeks and subsequently embedded in paraffin. Sagittal paraffin sections (4 μm thick) were prepared and stained with Hematoxylin and Eosin (H&E) and Masson’s trichrome to visualize the bone morphology and maturation. Histological slides were evaluated using quantitative histomorphometry to assess new bone regeneration. The area of new bone formation within each defect was quantified using NIS-Elements D software (version 5.21.00; Nikon Metrology GmbH, Düsseldorf, Germany). All histological slides were independently evaluated by two investigators (T.L. and K.R.), who were unaware of the sample identity. Prior to analysis, both observers underwent training and calibration using predefined standardized evaluation criteria. Any discrepancies were jointly reviewed and resolved by consensus to ensure consistency of the assessment.

### Immunohistochemical analysis

Immunohistochemical analysis was performed on paraffin sections using an anti-osteocalcin (OCN) antibody (1:200 dilution; sc-30044, Santa Cruz Biotechnology, Texas, USA). Biotin (Cat# ab64256, Abcam, Cambridge, UK) and streptavidin complex (Cat# ab64269, Abcam, Cambridge, UK) were used as secondary detection reagents following the manufacturer’s protocol. Positive immunoreactivity was visualized with a DAB substrate kit (Cat# ab64238, Abcam, Cambridge, USA), followed by counterstaining with Mayer’s hematoxylin solution (Cat# 05–06002, Bio-optica, Milano, Italy). Negative controls were performed by omitting the primary antibody. Osteocalcin-positive cells were quantified and expressed as the number of positively stained cells per high-power field (HPF) using images acquired with a 20 × objective lens on a Nikon Eclipse Ts2R microscope equipped with a DS-Fi3 digital camera. Quantitative analysis was performed on three randomly selected areas per HPF, defined as the field of view at 20 × magnification, corresponding to an area of approximately 0.95 mm^2^ per HPF. Image acquisition and analysis were conducted using NIS-Elements D software (version 5.21.00; Nikon Metrology GmbH, Düsseldorf, Germany). All histological slides were independently examined by two investigators (T.L. and K.R.), who were unaware of the sample identity.

### Tartrate-resistant acid phosphatase (TRAP) staining

Paraffin sections (4 μm thick) were deparaffined and rehydrated, then incubated in pre-warmed TRAP staining solution (Sigma-Aldrich, Steinheim, Germany), comprising TRAP basic incubation medium, Fast Red Violet LB Salt (Sigma-Aldrich, Steinheim, Germany), and freshly prepared naphthol AS-MX phosphate substrate (Sigma-Aldrich, Steinheim, Germany), at 37 °C in water bath for 30 minutes. Sections were subsequently counterstained with 0.02% fast green for 10 minutes. Multinucleated TRAP-positive cells, indicated by red-violet staining, were quantified under a light microscope (Nikon, Japan) by two investigators (T.L. and K.R.), who were blinded to the sample identities.

### Micro-computed tomography (Micro-CT)

New bone formation within the defect site was evaluated using three-dimensional micro-computed tomography (ultra-high resolution micro-CT; model; UHR U-CT, MILabs, Houten, Netherlands) at 4 and 12 weeks post-implantation. The X-ray tube was operated at 50 kV and 0.24 mA. The acquired 3D data were reconstructed using MILabs reconstruction software (MILabs, Houten, The Netherlands) using a voxel size of 17 μm. Bone regeneration within the calvarial defects was quantified by the ratio of bone volume to tissue volume (BV/TV), while residual graft material was quantified by the ratio of bone substitute volume to tissue volume (BSV/TV), using Imalytics Preclinical software (version 3.0, Gremse-IT, Aachen, Germany).

To quantify the volumes of newly formed bone and residual graft material, the region of interest (ROI) was defined as a cylindrical volume corresponding to the defect site, with a diameter of 5 mm and a height encompassing the entire grafted specimen. The total ROI volume was calculated using the standard geometric formula $V=\pi r^{\text{2}}h$, where 𝑟 represents the radius of the defect (2.5 mm) and ℎ denotes the measured height of the grafted area. The grayscale histogram of each scan was analyzed to identify peaks corresponding to newly mineralized bone and graft particles. Based on these analyses, specific threshold values were applied to segment new bone and residual graft from the surrounding old bone. Subsequently, manual correction was performed to eliminate surrounding tissues and imaging artifacts. The resulting segmented volumes were used to calculate the relative percentages of newly formed bone and residual grafting material within the defect.

### Statistical analysis

Statistical analyses were performed using GraphPad Prism 9 (GraphPad Software Inc., San Diego, CA, USA). A p-value < 0.05 was considered statistically significant. Data are expressed as mean ± standard error of the mean (SEM). Sample sizes were determined based on previous rat critical-size calvarial defect studies, in which histomorphometric analyses were commonly performed using approximately 5–10 defects per group. In the present bilateral defect design, each calvarial defect was defined a priori as an independent experimental unit, resulting in nine defects per material group at each time point, which was considered sufficient to detect biologically relevant differences typically observed in preclinical bone regeneration studies [40–44].

The Shapiro-Wilks test was used to assess the normality of the data. For normally distributed data, including BV/TV and BSV/TV, one-way analysis of variance (ANOVA) followed by Bonferroni multiple comparison test was used. Non-normally distributed data, including the percentage of new bone formation in the defect area, number of osteoblasts, osteocytes and osteoclasts were analyzed using non-parametric tests, including the Kruskal-Wallis test followed by Dunn’s post-hoc analysis.

## Results

**Fig 3:**
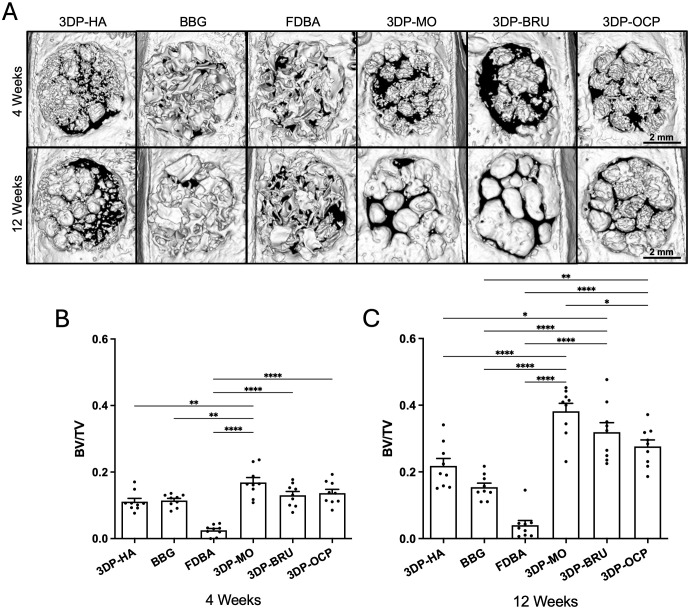
(A) Representative micro-CT 3D images at 4 and 12 weeks post-implantation.Scale bar = 2 mm. (B&C) New bone formation within the defect site as quantified by BV/TV. (C&E) Residual bone graft as quantified by BSV/TV. N = 9 per group. The data are expressed as mean ± standard error of the mean (SEM). (*p < 0.05, **p < 0.01, ***p < 0.001, ****p < 0.0001); one-way ANOVA followed by Bonferroni multiple comparison test.

To evaluate new bone formation within the calvarial defect, micro-CT analysis was performed at 4 and 12 weeks post-implantation (Fig 3). At 4 weeks, the 3DP-MO group (0.1689 ± 0.0149, n = 9) exhibited significantly greater new bone formation than the 3DP-HA (0.1112 ± 0.0099, *p* = 0.0040, n = 9), BBG (0.1146 ± 0.0063, *p* = 0.0083, n = 9), and FDBA (0.0251 ± 0.0054, *p* < 0.0001, n = 9) groups, as indicated by the BV/TV ratio (Fig 3B, S1-S2 Tables). Additionally, new bone formation in the 3DP-BRU (0.1305 ± 0.0113, n = 9) and 3DP-OCP (0.1367 ± 0.0116, n = 9) groups was significantly higher than that in the FDBA group (*p* < 0.0001).

**Fig 4:**
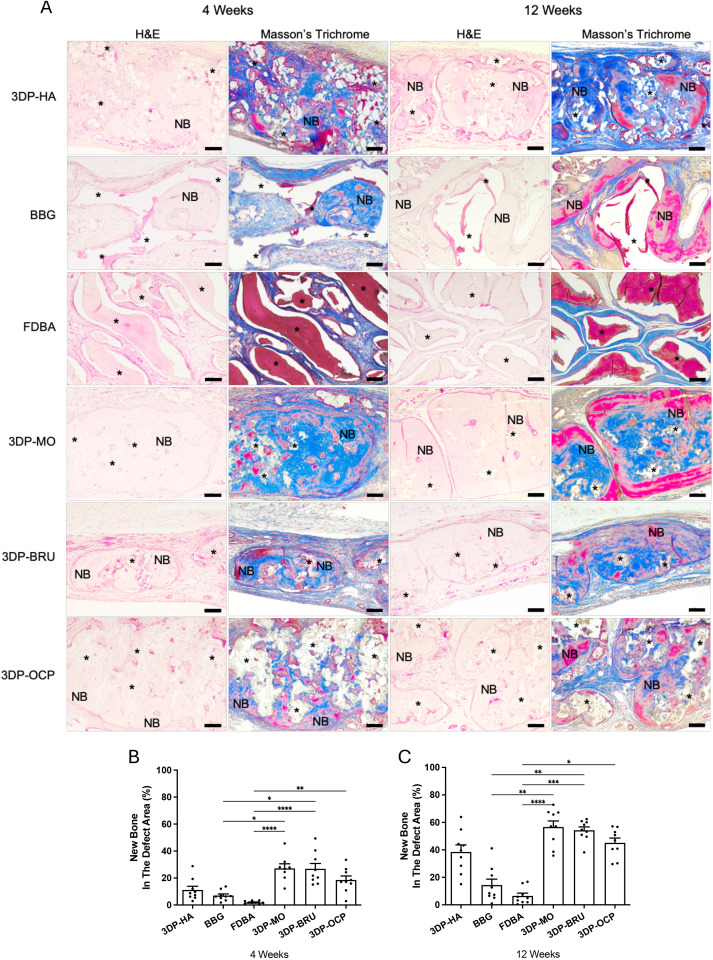
(A) Decalcified histology of calvarial defect at 4 and 12 weeks post-implantation stained with H&E and Masson’s trichrome.NB indicates new bone. Asterisks (*) indicates implanted material residues. Scale bar = 100 μm. (B&C) Quantitative analysis of new bone formation area (%). N = 8-9 per group. The data are expressed as mean ± standard error of the mean (SEM). (*p < 0.05, **p < 0.01, ***p < 0.001, ****p < 0.0001); Kruskal-Wallis test followed by Dunn’s post-hoc test.

At 12 weeks, the amount of newly formed bone within the defect site was significantly greater in the 3DP-MO (0.3819 ± 0.0239, n = 9) and 3DP-BRU (0.3193 ± 0.0284, n = 9) groups than in the 3DP-HA group (0.2179 ± 0.0223, n = 9) (*p* < 0.0001 and *p* = 0.0181, respectively). Both groups also exhibited significantly greater bone formation than BBG (0.1543 ± 0.0121, n = 9) and FDBA (0.0403 ± 0.0142, n = 9) (*p* < 0.0001 for both comparison). Additionally, 3DP-MO exhibited significantly greater new bone formation than 3DP-OCP (0.2763 ± 0.0195, n = 9; *p* = 0.0118). Moreover, 3DP-OCP revealed significantly increased bone regeneration compared with BBG (*p* = 0.0021) and FDBA (*p* < 0.0001) (Fig 3C, S3-S4 Tables). These findings were consistent with the histological analysis. Fig 4A shows representative calvarial tissue sections stained with H&E and Masson’s trichrome. No evidence of graft rejection was observed, as indicated by the absence of immune cell infiltration in the implant area. New bone ingrowth into the defect was observed across all groups, suggesting good biocompatibility and osteoconductivity. However, BBG and FDBA exhibited noticeably less new bone formation than the other materials. At both 4 and 12 weeks post-implantation, 3DP-MO (4 weeks: 27.10 ± 3.40%, n = 8; 12 weeks: 56.65 ± 4.43%, n = 9) and 3DP-BRU (4 weeks: 26.85 ± 3.97%, n = 9; 12 weeks: 54.26 ± 2.51%, n = 9) showed significantly greater bone ingrowth into the scaffolds than BBG (4 weeks: 6.99 ± 1.27%, n = 9; 12 weeks: 14.43 ± 4.27%, n = 9) (4 weeks: *p* = 0.0156 and *p* = 0.0250, respectively; 12 weeks: *p* = 0.0011 and *p* = 0.0032, respectively). In addition, 3DP-MO, 3DP-BRU, and 3DP-OCP (4 weeks: 18.49 ± 2.97%, n = 9; 12 weeks: 45.11 ± 3.48%, n = 9) demonstrated significantly greater new bone formation than FDBA (4 weeks: 1.93 ± 0.37%, n = 9; 12 weeks: 6.50 ± 2.00%, n = 9) (4 weeks: *p* < 0.0001, *p* < 0.0001, and *p* = 0.0037, respectively; 12 weeks: *p* < 0.0001, *p* = 0.0001, and *p* = 0.0125, respectively) (Fig 4B and 4C, S9-S12 Tables).

**Fig 5:**
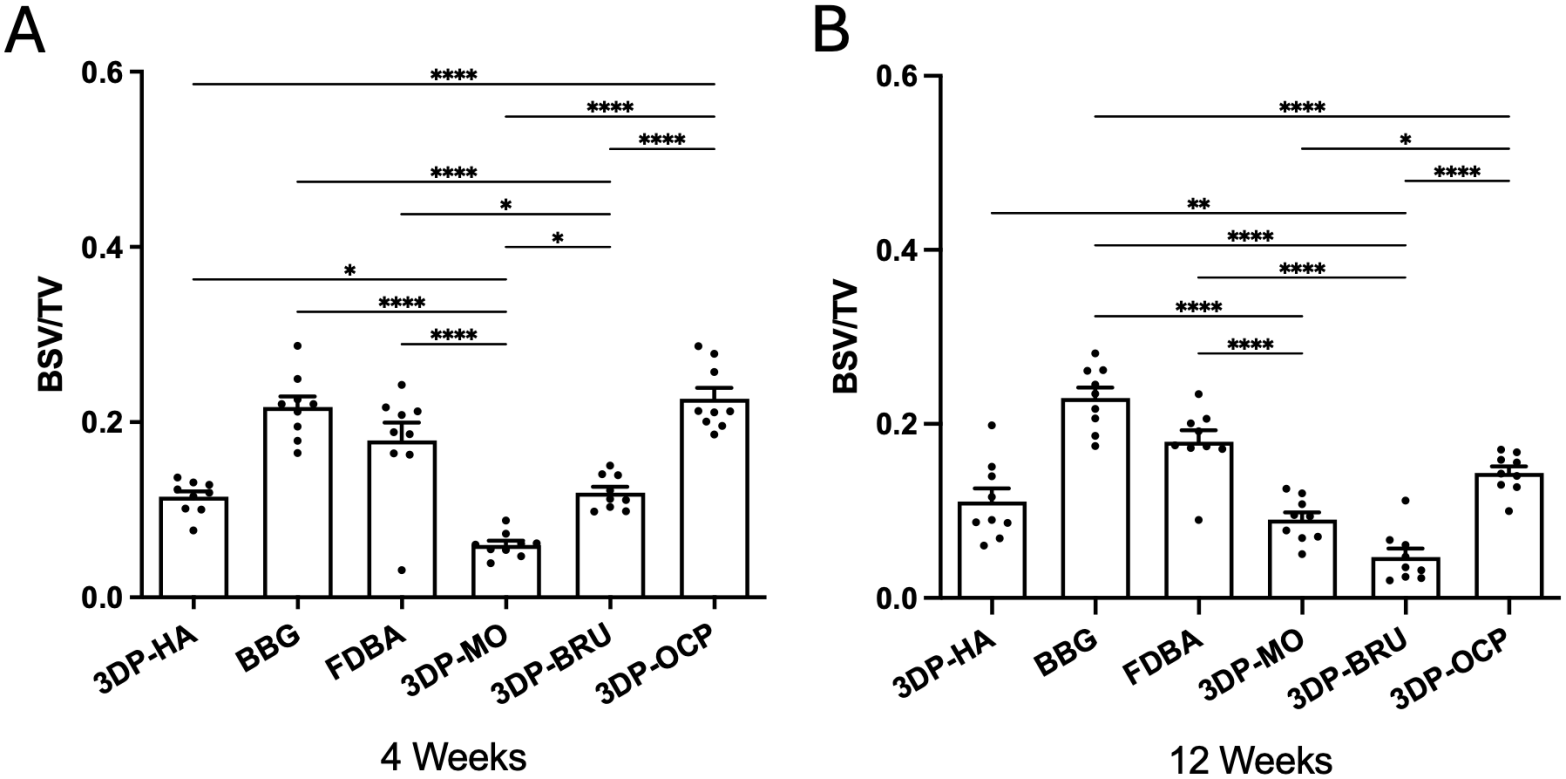
Residual bone graft as quantified by BSV/TV. N = 9 per group. The data are expressed as mean ± standard error of the mean (SEM). (*p < 0.05, **p < 0.01, ***p < 0.001, ****p < 0.0001); one-way ANOVA followed by Bonferroni multiple comparison test.

To assess resorbability over time, residual bone graft within the defect was quantified using BSV/TV ratio. At 4 weeks post-implantation, the 3DP-MO group (0.0598 ± 0.0047, n = 9) exhibited a significantly lower amount of residual graft than the 3DP-HA (0.1148 ± 0.0063, n = 9; *p* = 0.0264), BBG (0.2172 ± 0.0122, n = 9; *p* < 0.0001), and FDBA (0.1793 ± 0.0204, n = 9; *p* < 0.0001) groups. Similarly, the 3DP-BRU group (0.1196 ± 0.0066, n = 9) showed significantly less residual graft than BBG (*p* < 0.0001) and FDBA (*p* = 0.0113) (Fig 5A, S5-S6 Tables). At 12 weeks, 3DP-MO (0.0900 ± 0.0084, n = 9) demonstrated significantly greater resorption than BBG (0.2298 ± 0.0120, n = 9) and FDBA (0.1795 ± 0.0131, n = 9) (*p* < 0.0001 for both comparisons). The 3DP-BRU group (0.0469 ± 0.0098, n = 9) also showed significantly less residual graft than 3DP-HA (0.1108 ± 0.0150, n = 9; *p* = 0.0033), BBG (0.2298 ± 0.0120, n = 9; *p* < 0.0001), and FDBA (0.1795 ± 0.0131, n = 9; *p* < 0.0001). Moreover, 3DP-OCP (0.1436 ± 0.0075, n = 9) exhibited significantly greater resorption than BBG (*p* < 0.0001) (Fig 5B, S7-S8 Tables). Notably, 3DP-MO displayed higher resorbability than 3DP-BRU at 4 weeks (*p* = 0.0113) and greater resorbability than 3DP-OCP at both 4 weeks (0.2267 ± 0.0124, n = 9; *p* < 0.0001) and 12 weeks (0.1436 ± 0.0075, n = 9; p < 0.0237). Additionally, 3DP-BRU also demonstrated higher resorbability than 3DP-OCP at both time points (*p* < 0.0001).

### Resorbable 3D-printed calcium phosphate scaffolds promoted bone cells activity

**Fig 6:**
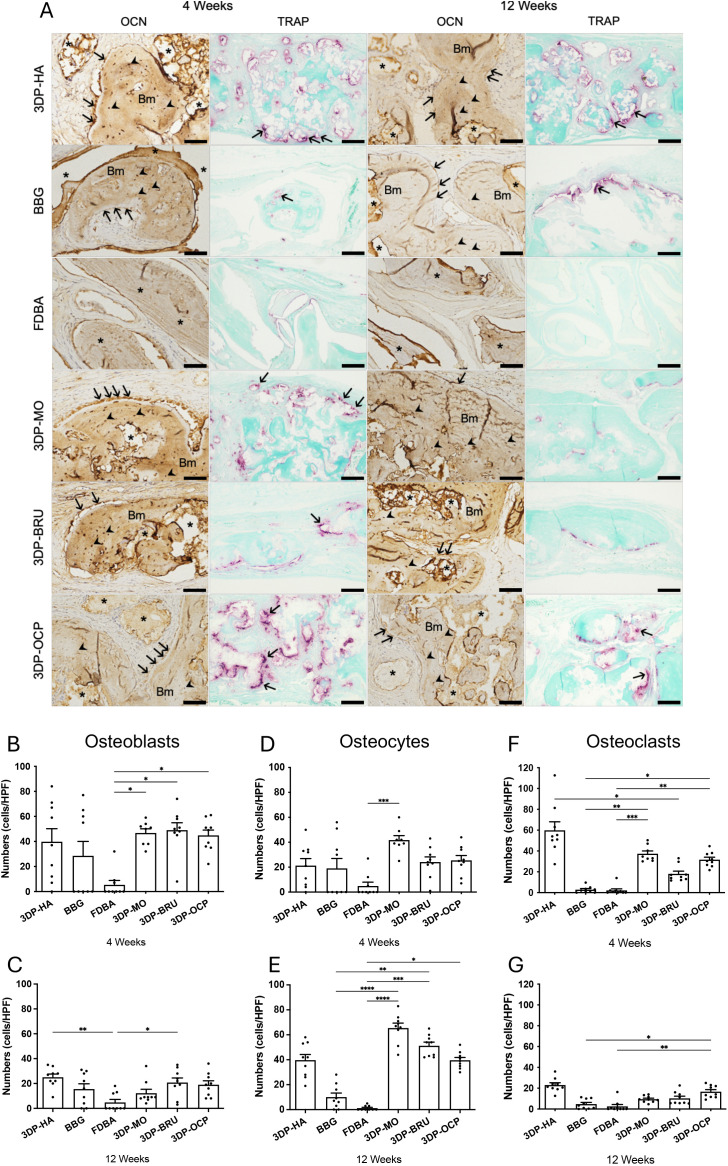
(A) The positive staining of osteocalcin and TRAP at 4 and 12 weeks post-implantation.Bm indicates bone matrix. Asterisks (*) indicates implanted material. Arrows indicates osteocalcin-positive osteoblasts and TRAP-positive osteoclasts. Arrowhead indicates osteocalcin-positive osteocytes. Scale bar = 50 μm for osteocalcin and 100 μm for TRAP stain. (B&C) Number of osteoblasts positive staining cells. (D&E) Number of osteocytes positive staining cells. (F&G) Number of osteoclasts positive staining cells. N = 8-9 per group. The data are expressed as mean ± standard error of the mean (SEM). (*p < 0.05, **p < 0.01, ***p < 0.001, ****p < 0.0001); Kruskal-Wallis test followed by Dunn’s post-hoc test.

Osteocalcin-positive staining was observed in newly regenerated bone within the calvarial defects. At 4 weeks post-implantation, the 3DP-MO (46.75 ± 3.35 cells/HPF, n = 8; *p* = 0.0385), 3DP-BRU (49.00 ± 5.93 cells/HPF, n = 9; *p* = 0.0122), and 3DP-OCP (44.89 ± 4.27 cells/HPF, n = 9; *p* = 0.0392) groups showed significantly higher numbers of osteocalcin-positive osteoblasts than the FDBA group (5.33 ± 3.53 cells/HPF, n = 9). At 12 weeks, both the 3DP-HA (25.00 ± 2.64 cells/HPF, n = 9; *p* = 0.0022) and 3DP-BRU (20.78 ± 3.71 cells/HPF, n = 9; *p* = 0.0472) groups exhibited significantly increased numbers of osteocalcin-positive osteoblasts compared with FDBA (4.78 ± 2.45 cells/HPF, n = 9) (Fig 6B, S13-S14 Tables and 6C, S15-S16 Tables).

A significantly higher number of osteocyte-positive cells was observed at 4 weeks post-implantation only in the 3DP-MO group (41.75 ± 3.52 cells/HPF, n = 8; *p* = 0.0003) compared with FDBA (4.89 ± 3.08 cells/HPF, n = 9). At 12 weeks, the 3DP-MO (65.44 ± 3.91 cells/HPF, n = 9; *p* < 0.0001), 3DP-BRU (51.22 ± 2.90 cells/HPF, n = 9; *p* = 0.0002), and 3DP-OCP (39.56 ± 2.27 cells/HPF, n = 9; *p* = 0.0382) groups revealed significantly higher numbers of osteocyte-positive cells than FDBA (1.22 ± 0.64 cells/HPF, n = 9). Moreover, the 3DP-MO (*p* < 0.0001) and 3DP-BRU (*p* = 0.0058) groups demonstrated significantly greater numbers of osteocyte-positive cells than BBG (10.00 ± 3.24 cells/HPF, n = 9) (Fig 6D, S17-S18 Tables and 6E, S19-S20 Tables).

TRAP staining was performed to evaluate osteoclast activity. At 4 weeks post-implantation, the 3DP-MO (37.33 ± 2.73 cells/HPF, n = 8) and 3DP-OCP (31.67 ± 2.39 cells/HPF, n = 9) groups showed significantly higher numbers of TRAP-positive multinucleated cells than BBG (2.89 ± 1.01 cells/HPF, n = 9; *p* = 0.0030 and *p* = 0.0275, respectively) and FDBA group (2.19 ± 1.50 cells/HPF, n = 9; *p* = 0.0006 and *p* = 0.0068, respectively). In addition, 3DP-HA (58.89 ± 8.16 cells/HPF, n = 9) showed a significantly greater number of TRAP-positive cells than 3DP-BRU (18.07 ± 2.61 cells/HPF, n = 9; *p* = 0.0297). At 12 weeks post-implantation, the 3DP-OCP group (16.67 ± 1.99 cells/HPF, n = 9) demonstrated significantly higher numbers of TRAP-positive cells than BBG (4.78 ± 1.57 cells/HPF, n = 9; *p* = 0.0233) and FDBA (2.59 ± 1.80 cells/HPF, n = 9; *p* = 0.0013) (Fig 6F, S21-S22 Tables and 6G, S23-S24 Tables).

## Discussion

This study indicates that all 3D-printed samples and commercial bone grafts are biocompatible, osteoconductive, and capable of supporting bone regeneration without inducing systemic or local adverse effects. This investigation is the preliminary study to see the efficacy of 3D-printed calcium phosphates in a granular form without utilizing the full design flexibility of 3D printing to produce anatomically complex or patient-specific implants. However, the findings support the potential for broader application of binder jetting as a scalable and adaptable technique for producing synthetic bone graft substitutes. Future studies could explore how this technology can be leveraged for fabricating custom-shaped implants to better match defect geometries in clinical scenarios, thereby maximizing the benefits of additive manufacturing in bone tissue engineering.

From micro-CT and histological analysis, the resorbable 3D-printed calcium phosphate scaffolds exhibited significantly greater new bone formation than the control materials, particularly BBG and FDBA, at both 4 and 12 weeks post-implantation. In contrast, implantation with BBG and FDBA produced only limited new bone formation, and the defect sites remained predominantly occupied by residual grafting materials up to 12 weeks post-implantation. This observation may reflect the characteristically slow degradation rates of these materials in a contained defect model.

Previous studies have reported that BBG exhibits significantly slow resorption in humans, remaining at the implantation site for up to 4 years [45]. Similarly, FDBA has been shown to resorb more slowly than autologous bone graft following 5-years of atrophic maxillary reconstruction [46]. While this slow resorption is often suggested as an advantage when combined with autologous bone graft to compensate for volume loss [47], it appears to have acted as a physical barrier to new bone volume accumulation in the present study.

BRU, MO, and OCP are soluble calcium phosphates with documented osteoconductive potential to enhance bone regeneration, as confirmed in various reports [7,48,49]. Their solubility at 25°C follows the order: BRU (~0.088 g/L)> MO (~0.048 g/L)> OCP (~0.0081 g/L) [50], which are much greater that of HA (~0.0003 g/L) [50] and are often used to suggest potential *in vivo* resorbability [51]. Previous studies have reported that a slow resorption rate of biomaterials *in vivo* can limit their effectiveness as bone grafts [15]. To the best of our knowledge, this is the first *in vivo* study directly comparing the three types of resorbable binder jet 3D-printed calcium phosphates.

Based on our findings, 3DP-MO exhibited progressive enhancement in bone regeneration, showing significantly greater efficacy than 3DP-OCP at 12 weeks post-implantation. At 4 weeks, 3DP-MO also demonstrated higher apparent resorbability than both 3DP-BRU and 3DP-OCP, while 3DP-BRU resorbed faster than 3DP-OCP. By 12 weeks, both 3DP-MO and 3DP-BRU continued to exhibit significantly greater resorbability compared with 3DP-OCP. These results align with previous *in vivo* studies demonstrating that implantation of MO in the rabbit tibia for 4 weeks resulted in greater infiltration of newly formed bone than faster resorption compared with BRU [11]. Similarly, in a rabbit femur defect model evaluated over 12 weeks, MO showed more new bone formation and faster resorption than BRU [52].

There are two primary mechanisms contributing to the degradation of ceramic biomaterials: cellular activity and passive dissolution [53]. Differences in solubility and resorption rates among biomaterials likely influence the success of bone graft [54]. Previous studies have reported that BRU undergoes rapid degradation during the first week post-implantation, driven by simple dissolution, disintegration, and cellular activity [11,55,56]. Thereafter, the transformation of BRU into a less soluble crystalline apatite has been observed after 24 weeks post-implantation in sheep [57], which is thought to lead to a reduction in the resorption of the remaining material [58]. The degradation mechanism of MO is similarly suggested to involve both cellular activity and passive dissolution [53]. However, MO has been reported to exhibit a faster resorption rate *in vivo* despite having a lower solubility than BRU. Previous studies have shown that MO degraded without undergoing a phase transformation *in vivo* and demonstrates superior resorption compared with other calcium phosphate-based materials, including HA, TCP, and BRU [59]. Furthermore, MO is considered an ideal bone graft material due to its observed ability to maintain a balance between resorption and new bone formation [18,19,51].

OCP is widely recognized as a precursor to biological apatite [60] owing to its structural similarity to that HA [20]. Unlike more soluble calcium phosphate phases, OCP resorption is thought to occur predominantly through osteoclast-mediated cellular activity rather than simple physicochemical dissolution [61]. Moreover, OCP can gradually transform into HA both in vitro and in vivo, further contributing to its relative structural persistence [44]. The present study demonstrated that OCP exhibited the slowest resorption rate among the three types of resorbable 3D-printed calcium phosphate, comparable to HA at 12 weeks post-implantation. In the context of a contained calvarial defect, this slower, cell-mediated remodeling may not provide sufficiently rapid space generation to synchronize with the rate of new bone formation observed with MO and BRU. In such a stable geometric environment, where the surrounding calvarial bone prevents soft tissue collapse, the rapid dissolution of MO and BRU appears to facilitate more timely space provision, thereby better accommodating early bone ingrowth. In contrast, the prolonged structural stability of OCP, similar to that of 3DP-HA, BBG, and FDBA, may result in a kinetic mismatch between scaffold resorption and bone formation, possibly limiting the available space for tissue regeneration.

Although rapid scaffold resorption is sometimes considered a limitation due to the risk of volume loss, it did not appear detrimental in contained craniofacial defects where surrounding bone walls provide intrinsic space maintenance. However, in large non-contained or load-bearing defects, slower-degrading scaffolds or composite systems with enhanced mechanical stability may be required to maintain defect volume and mechanical integrity [62–64].

At the molecular level, osteocalcin (OCN) served as a marker of osteoblast and osteocyte activity, consistent with its role in matrix synthesis and mineralization [65,66]. Likewise, TRAP is used to identify osteoclasts, reflecting active bone resorption [67]. Together, these markers provided insight into the dynamic interplay between bone-forming and bone-resorbing cells within the healing environment. Histological analysis revealed increased numbers of osteocalcin-positive osteoblasts and osteocytes in defects treated with resorbable 3D-printed calcium phosphate scaffolds, particularly in the 3DP-MO group, which is consistent with its enhanced bone regeneration profile. TRAP staining indicated active osteoclast presence at 4 weeks in defects treated with resorbable 3D-printed scaffolds, particularly in 3DP-HA, 3DP-MO, and 3DP-OCP.

An apparent discrepancy between molecular and morphometric outcomes was observed in some groups. For example, the 3DP-HA group demonstrated increased OCN expression despite relatively low BV/TV values, indicating that matrix maturation and mineralized tissue accumulation do not necessarily occur in parallel. Because OCN reflects late-stage osteoblastic differentiation, its elevation likely represents ongoing maturation of newly formed bone rather than a substantial increase in bone volume. Thus, indicators of cellular activity and bone quality should be interpreted independently from volumetric measurements. Notably, TRAP-positive cells remained elevated in the 3DP-HA and 3DP-OCP groups at 12 weeks, suggesting the possibility of sustained osteoclast-mediated remodeling associated with slower-resorbing materials. This observation may be attributed to their resorption mechanism, primarily driven by osteoclastic activity rather than simple physicochemical dissolution [61]. However, elevated TRAP activity might not correspond to greater apparent scaffold resorption compared with the 3DP-MO group. This observation highlights that the presence of osteoclasts and volumetric material loss are related but not directly proportional phenomena. TRAP staining reflects osteoclast recruitment and surface remodeling activity rather than bulk degradation efficiency. Sustained TRAP positivity in slower-resorbing groups likely indicates prolonged cell-mediated surface remodeling associated with persistent material presence rather than accelerated scaffold resorption.

Additionally, residual graft material and newly formed bone often exhibit comparable radiodensity on micro-CT, potentially leading to segmentation inaccuracies and partial misclassification. This limitation may influence BV/TV quantification and obscure the relationship between osteoclast activity and apparent scaffold degradation. Together, these findings underscore that osteoclast activity, material resorption, and bone formation are interrelated yet temporally distinct processes. Accurate interpretation of long-term remodeling, therefore, requires integration of histological and imaging data, reinforcing the importance of scaffold degradation kinetics in modulating regenerative outcomes.

Apart from resorbability alone, the different regenerative outcomes observed among the groups also highlight a complex interplay between initial scaffold architecture and material transformation kinetics. The 3DP-MO group exhibited the most robust bone formation, potentially due to a synergistic effect between its high initial porosity and its rapid resorption rate. While high initial porosity provides an immediate scaffold for cellular infiltration, the critical role of resorption-driven space provision is further underscored by the 3DP-BRU group. Despite possessing the lowest initial porosity, the 3DP-BRU scaffolds achieved superior bone volume compared to the more stable 3DP-OCP and 3DP-HA groups. This suggests that in this study model, the capacity of a material to actively produce a space through dissolution appears to be a more vital determinant of new bone volume than initial void space or inherent osteoconductivity alone.

From a fabrication perspective, binder jetting provides a scalable route for producing calcium phosphate scaffolds; however, phase homogeneity depends on solution diffusion during post-printing transformation. Microstructural heterogeneities inherent to powder-bed processing, including intra-strut porosity or minor residual binder traces, may subtly influence local degradation kinetics. Although previous validation confirmed high phase purity and biocompatibility using the employed protocols [68], high-resolution characterization would further clarify how microstructural variations affect long-term remodeling behavior.

The limitations of this study include the use of a rat calvarial defect model, which represents a non-load-bearing site and may not fully reflect the biological and mechanical conditions of load-bearing bones encountered in clinical applications. Although an empty-defect control was not included, the defect size used (5 mm in diameter) has been widely validated as a critical-sized defect with minimal spontaneous healing [40,42,43,69]. Accordingly, the contribution of spontaneous healing to the observed outcomes is expected to be minimal, although this limitation should be considered when interpreting the results. Sterilization procedures may also influence early biological responses. All scaffolds were sterilized by gamma irradiation (25 kGy), a clinically standard dose. While inorganic calcium phosphates materials are generally considered resistant to radiation-induced structural changes [70,71], minor surface modifications affecting early protein adsorption and cell attachment cannot be entirely excluded. In addition, gamma irradiation at this dose is not expected to substantially alter the bulk mechanical integrity of ceramic scaffolds, although subtle surface-level effects may occur. Therefore, although the observed differences in regeneration are primarily attributable to intrinsic material properties, potential surface-level effects associated with sterilization warrant further investigation. In addition, the specific biological mechanisms responsible for the enhanced bone regeneration, the detailed resorption processes, and the correlation between scaffold resorption and new bone formation were not investigated. Future studies should therefore employ load-bearing animal models and include mechanistic analyses to validate the clinical relevance of these findings and elucidate the role of calcium phosphate resorbability in bone regeneration. Further optimization of scaffold composition and structure should also be undertaken to maximize regenerative performance.

## Conclusion

In conclusion, resorbable binder jet 3D-printed calcium phosphate scaffolds, including 3DP-MO, 3DP-BRU, and 3DP-OCP, demonstrated enhanced bone regeneration compared with 3DP-HA and commercial allograft and xenograft materials in the rat calvarial defect model. While improved outcomes were observed, the underlying mechanisms were not directly investigated in this study; therefore, the superior bone regeneration cannot be conclusively attributed to specific physicochemical properties such as solubility. Among the evaluated scaffolds, 3DP-MO demonstrated the most balanced profile between degradation and new bone formation. These findings highlight the potential of compositionally tailored 3D-printed calcium phosphate scaffolds as promising bone graft substitutes with tunable osteoconductivity, osteogenic potential, and resorbability, although further mechanistic and physicochemical investigations in clinically relevant models are required to confirm their translational applicability.

## Supporting information

S1 Table Quantitative micro-CT analysis (BV/TV ratio) at 4 weeks. (DOCX)

S2 Table Statistical comparisons of BV/TV ratio at 4 weeks. (DOCX)

S3 Table Quantitative micro-CT analysis of BV/TV ratio at 12 weeks. (DOCX)

S4 Table Statistical comparisons of BV/TV ratio at 12 weeks. (DOCX)

S5 Table Quantitative micro-CT analysis of BSV/TV ratio at 4 weeks. (DOCX)

S6 Table Statistical comparisons of BSV/TV ratio at 4 weeks. (DOCX)

S7 Table Quantitative micro-CT analysis of BSV/TV ratio at 12 weeks. (DOCX)

S8 Table Statistical comparisons of BSV/TV ratio at 12 weeks. (DOCX)

S9 Table Quantitative percent of new bone in the defect area analysis at 4 weeks. (DOCX)

S10 Table Statistical comparisons of quantitative percent of new bone in the defect area analysis at 4 weeks. (DOCX)

S11 Table Quantitative percent of new bone in the defect area analysis at 12 weeks. (DOCX)

S12 Table Statistical comparisons of quantitative percent of new bone in the defect area analysis at 12 weeks. (DOCX)

S13 Table Quantitative number of osteoblasts at 4 weeks. (DOCX)

S14 Table Statistical comparisons of quantitative number of osteoblasts at 4 weeks. (DOCX)

S15 Table Quantitative number of osteoblasts at 12 weeks. (DOCX)

S16 Table Statistical comparisons of quantitative number of osteoblasts at 12 weeks. (DOCX)

S17 Table Quantitative number of osteocytes at 4 weeks. (DOCX)

S18 Table Statistical comparisons of quantitative number of osteocytes at 4 weeks. (DOCX)

S19 Table Quantitative number of osteocytes at 12 weeks. (DOCX)

S20 Table Statistical comparisons of quantitative number of osteocytes at 12 weeks. (DOCX)

S21 Table Quantitative number of TRAP positive cells at 4 weeks. (DOCX)

S22 Table Statistical comparisons of quantitative number of TRAP positive cells at 4 weeks. (DOCX)

S23 Table Quantitative number of TRAP positive cells at 12 weeks. (DOCX)

S24 Table Statistical comparisons of quantitative number of TRAP positive cells at 12 weeks. (DOCX)
