# Supplementary material for: In vivo evaluation of binder jet 3D-Printed monetite, brushite, and octacalcium phosphate: A comparative study for bone regeneration in a rat calvarial defect model
Source: PLoS One. 2026 May 15;21(5):e0349259. doi: 10.1371/journal.pone.0349259 (PMC13178867; doi:10.1371/journal.pone.0349259)
Supplement: S3 Table — (DOCX) [file pone.0349259.s003.docx]

**S3 Table Quantitative micro-CT analysis of BV/TV ratio at 12 weeks**

| **Group** | **Mean** | **SEM** | **n** |
| --- | --- | --- | --- |
| 3DP-HA | 0.2179 | 0.0223 | 9 |
| BBG | 0.1543 | 0.0121 | 9 |
| FDBA | 0.0403 | 0.0142 | 9 |
| 3DP-MO | 0.3819 | 0.0239 | 9 |
| 3DP-BRU | 0.3193 | 0.0284 | 9 |
| 3DP-OCP | 0.2763 | 0.0195 | 9 |

*Data are presented as mean ± SEM (n = 9 per group). BV/TV was analyzed using one-way ANOVA followed by Bonferroni multiple comparisons test.*
