# Supplementary material for: In vivo evaluation of binder jet 3D-Printed monetite, brushite, and octacalcium phosphate: A comparative study for bone regeneration in a rat calvarial defect model
Source: PLoS One. 2026 May 15;21(5):e0349259. doi: 10.1371/journal.pone.0349259 (PMC13178867; doi:10.1371/journal.pone.0349259)
Supplement: S5 Table — (DOCX) [file pone.0349259.s005.docx]

**S5 Table Quantitative micro-CT analysis of BSV/TV ratio at 4 weeks**

| **Group** | **Mean** | **SEM** | **n** |
| --- | --- | --- | --- |
| 3DP-HA | 0.1148 | 0.0063 | 9 |
| BBG | 0.2172 | 0.0122 | 9 |
| FDBA | 0.1793 | 0.0204 | 9 |
| 3DP-MO | 0.0598 | 0.0047 | 9 |
| 3DP-BRU | 0.1196 | 0.0066 | 9 |
| 3DP-OCP | 0.2267 | 0.0124 | 9 |

*Data are presented as mean ± SEM (n = 9 per group). Statistical analysis was performed using one-way ANOVA followed by Bonferroni multiple comparisons test.*
