# Supplementary material for: In vivo evaluation of binder jet 3D-Printed monetite, brushite, and octacalcium phosphate: A comparative study for bone regeneration in a rat calvarial defect model
Source: PLoS One. 2026 May 15;21(5):e0349259. doi: 10.1371/journal.pone.0349259 (PMC13178867; doi:10.1371/journal.pone.0349259)
Supplement: S8 Table — (DOCX) [file pone.0349259.s008.docx]

**S8 Table Statistical comparisons of BSV/TV ratio at 12 weeks**

| **Comparison** | **Summary** | **Adjusted p Value** |
| --- | --- | --- |
| 3DP-HA vs. 3DP-MO | ns | >0.9999 |
| 3DP-HA vs. 3DP-BRU | ** | 0.0033 |
| 3DP-HA vs. 3DP-OCP | ns | 0.6856 |
| BBG vs. 3DP-MO | **** | <0.0001 |
| BBG vs. 3DP-BRU | **** | <0.0001 |
| BBG vs. 3DP-OCP | **** | <0.0001 |
| FDBA vs. 3DP-MO | **** | <0.0001 |
| FDBA vs. 3DP-BRU | **** | <0.0001 |
| FDBA vs. 3DP-OCP | ns | 0.4464 |
| 3DP-MO vs. 3DP-BRU | ns | 0.1447 |
| 3DP-MO vs. 3DP-OCP | * | 0.0237 |
| 3DP-BRU vs. 3DP-OCP | **** | <0.0001 |
| **Effect size** (Partial eta squared; $\eta_{p}^{2}$) = 0.78 | | |

*Statistical analysis was performed using one-way ANOVA followed by Bonferroni multiple comparisons test.*
