# Supplementary material for: In vivo evaluation of binder jet 3D-Printed monetite, brushite, and octacalcium phosphate: A comparative study for bone regeneration in a rat calvarial defect model
Source: PLoS One. 2026 May 15;21(5):e0349259. doi: 10.1371/journal.pone.0349259 (PMC13178867; doi:10.1371/journal.pone.0349259)
Supplement: S9 Table — (DOCX) [file pone.0349259.s009.docx]

**S9 Table Quantitative percent of new bone in the defect area analysis at 4 weeks**

| **Group** | **Mean (%)** | **SEM** | **n** |
| --- | --- | --- | --- |
| 3DP-HA | 11.17 | 2.70 | 9 |
| BBG | 6.99 | 1.27 | 9 |
| FDBA | 1.93 | 0.37 | 9 |
| 3DP-MO | 27.10 | 3.39 | 8 |
| 3DP-BRU | 26.85 | 3.97 | 9 |
| 3DP-OCP | 18.49 | 2.97 | 9 |

*Data are presented as mean ± SEM (n =8- 9 per group). Statistical analysis was performed using one-way ANOVA followed by Bonferroni multiple comparisons test.*

**One sample from the 3DP-MO group was excluded due to tissue processing artifacts.*
