# Supplementary material for: In vivo evaluation of binder jet 3D-Printed monetite, brushite, and octacalcium phosphate: A comparative study for bone regeneration in a rat calvarial defect model
Source: PLoS One. 2026 May 15;21(5):e0349259. doi: 10.1371/journal.pone.0349259 (PMC13178867; doi:10.1371/journal.pone.0349259)
Supplement: S11 Table — (DOCX) [file pone.0349259.s011.docx]

**S11 Table Quantitative percent of new bone in the defect area analysis at 12 weeks**

| **Group** | **Mean (%)** | **SEM** | **n** |
| --- | --- | --- | --- |
| 3DP-HA | 38.50 | 5.14 | 9 |
| BBG | 14.43 | 4.27 | 9 |
| FDBA | 6.50 | 2.00 | 9 |
| 3DP-MO | 56.65 | 4.43 | 9 |
| 3DP-BRU | 54.26 | 2.51 | 9 |
| 3DP-OCP | 45.11 | 3.48 | 9 |

*Data are presented as mean ± SEM (n = 9 per group). Statistical analysis was performed using one-way ANOVA followed by Bonferroni multiple comparisons test.*
