# Supplementary material for: In vivo evaluation of binder jet 3D-Printed monetite, brushite, and octacalcium phosphate: A comparative study for bone regeneration in a rat calvarial defect model
Source: PLoS One. 2026 May 15;21(5):e0349259. doi: 10.1371/journal.pone.0349259 (PMC13178867; doi:10.1371/journal.pone.0349259)
Supplement: S15 Table — (DOCX) [file pone.0349259.s015.docx]

**S15 Table Quantitative number of osteoblasts at 12 weeks**

| **Group** | **Mean (cells/HPF)** | **SEM** | **n** |
| --- | --- | --- | --- |
| 3DP-HA | 25.00 | 2.64 | 9 |
| BBG | 15.56 | 4.26 | 9 |
| FDBA | 4.78 | 2.45 | 9 |
| 3DP-MO | 12.22 | 3.13 | 9 |
| 3DP-BRU | 20.78 | 3.71 | 9 |
| 3DP-OCP | 19.00 | 3.17 | 9 |

*Data are presented as mean ± SEM (n = 9 per group). Statistical analysis was performed using one-way ANOVA followed by Bonferroni multiple comparisons test.*
