# Supplementary material for: In vivo evaluation of binder jet 3D-Printed monetite, brushite, and octacalcium phosphate: A comparative study for bone regeneration in a rat calvarial defect model
Source: PLoS One. 2026 May 15;21(5):e0349259. doi: 10.1371/journal.pone.0349259 (PMC13178867; doi:10.1371/journal.pone.0349259)
Supplement: S17 Table — (DOCX) [file pone.0349259.s017.docx]

**S17 Table Quantitative number of osteocytes at 4 weeks**

| **Group** | **Mean (cells/HPF)** | **SEM** | **n** |
| --- | --- | --- | --- |
| 3DP-HA | 21.22 | 5.69 | 9 |
| BBG | 19.00 | 7.95 | 9 |
| FDBA | 4.89 | 3.08 | 9 |
| 3DP-MO | 41.75 | 3.52 | 8 |
| 3DP-BRU | 24.11 | 4.12 | 9 |
| 3DP-OCP | 25.44 | 3.90 | 9 |

*Data are presented as mean ± SEM (n =8- 9 per group). Statistical analysis was performed using one-way ANOVA followed by Bonferroni multiple comparisons test.*

**One sample from the 3DP-MO group was excluded due to tissue processing artifacts.*
