# Supplementary material for: In vivo evaluation of binder jet 3D-Printed monetite, brushite, and octacalcium phosphate: A comparative study for bone regeneration in a rat calvarial defect model
Source: PLoS One. 2026 May 15;21(5):e0349259. doi: 10.1371/journal.pone.0349259 (PMC13178867; doi:10.1371/journal.pone.0349259)
Supplement: S18 Table — (DOCX) [file pone.0349259.s018.docx]

**S18 Table Statistical comparisons of quantitative number of osteocytes at 4 weeks**

| **Comparison** | **Summary** | **Adjusted p Value** |
| --- | --- | --- |
| 3DP-HA vs. 3DP-MO | ns | 0.2753 |
| 3DP-HA vs. 3DP-BRU | ns | >0.9999 |
| 3DP-HA vs. 3DP-OCP | ns | >0.9999 |
| BBG vs. 3DP-MO | ns | 0.0790 |
| BBG vs. 3DP-BRU | ns | >0.9999 |
| BBG vs. 3DP-OCP | ns | >0.9999 |
| FDBA vs. 3DP-MO | *** | 0.0003 |
| FDBA vs. 3DP-BRU | ns | 0.2763 |
| FDBA vs. 3DP-OCP | ns | 0.1731 |
| 3DP-MO vs. 3DP-BRU | ns | 0.6811 |
| 3DP-MO vs. 3DP-OCP | ns | 0.9932 |
| 3DP-BRU vs. 3DP-OCP | ns | >0.9999 |
| **Effect size** (Epsilon squared, ε²) = 0.36 | | |

*Data were analyzed using one-way ANOVA followed by Bonferroni multiple comparisons test.*

**S19** **Table** Quantitative number of osteocytes at 12 weeks

| **Group** | **Mean** | **SEM** | **n** |
| --- | --- | --- | --- |
| 3DP-HA | 39.67 | 4.57 | 9 |
| BBG | 10.00 | 3.24 | 9 |
| FDBA | 1.22 | 0.64 | 9 |
| 3DP-MO | 65.44 | 3.91 | 9 |
| 3DP-BRU | 51.22 | 2.90 | 9 |
| 3DP-OCP | 39.56 | 2.27 | 9 |

*Data are presented as mean ± SEM (n = 9 per group). Statistical analysis was performed using one-way ANOVA followed by Bonferroni multiple comparisons test.*

**S20 Table** Statistical comparisons of quantitative number of osteocytes at 12 weeks

| **Comparison** | **Summary** | **Adjusted p Value** |
| --- | --- | --- |
| 3DP-HA vs. 3DP-MO | ns | 0.2396 |
| 3DP-HA vs. 3DP-BRU | ns | >0.9999 |
| 3DP-HA vs. 3DP-OCP | ns | >0.9999 |
| BBG vs. 3DP-MO | **** | <0.0001 |
| BBG vs. 3DP-BRU | ** | 0.0058 |
| BBG vs. 3DP-OCP | ns | 0.4509 |
| FDBA vs. 3DP-MO | **** | <0.0001 |
| FDBA vs. 3DP-BRU | *** | 0.0002 |
| FDBA vs. 3DP-OCP | * | 0.0382 |
| 3DP-MO vs. 3DP-BRU | ns | >0.9999 |
| 3DP-MO vs. 3DP-OCP | ns | 0.1641 |
| 3DP-BRU vs. 3DP-OCP | ns | >0.9999 |
| **Effect size** (Epsilon squared, ε²) = 0.80 | | |

*Data were analyzed using one-way ANOVA followed by Bonferroni multiple comparisons test.*

**S21** **Table** Quantitative number of TRAP positive cells at 4 weeks

| **Group** | **Mean** | **SEM** | **n** |
| --- | --- | --- | --- |
| 3DP-HA | 59.89 | 8.16 | 9 |
| BBG | 2.89 | 1.01 | 9 |
| FDBA | 2.19 | 1.50 | 9 |
| 3DP-MO | 37.33 | 2.73 | 8 |
| 3DP-BRU | 18.07 | 2.61 | 9 |
| 3DP-OCP | 31.67 | 2.39 | 9 |

*Data are presented as mean ± SEM (n =8- 9 per group). Statistical analysis was performed using one-way ANOVA followed by Bonferroni multiple comparisons test.*

**One sample from the 3DP-MO group was excluded due to tissue processing artifacts.*

**S22** **Table** Statistical comparisons of quantitative number of TRAP positive cells at 4 weeks

| **Comparison** | **Summary** | **Adjusted p Value** |
| --- | --- | --- |
| 3DP-HA vs. 3DP-MO | ns | >0.9999 |
| 3DP-HA vs. 3DP-BRU | * | 0.0297 |
| 3DP-HA vs. 3DP-OCP | ns | >0.9999 |
| BBG vs. 3DP-MO | ** | 0.0030 |
| BBG vs. 3DP-BRU | ns | >0.9999 |
| BBG vs. 3DP-OCP | * | 0.0275 |
| FDBA vs. 3DP-MO | *** | 0.0006 |
| FDBA vs. 3DP-BRU | ns | 0.4095 |
| FDBA vs. 3DP-OCP | ** | 0.0068 |
| 3DP-MO vs. 3DP-BRU | ns | 0.7569 |
| 3DP-MO vs. 3DP-OCP | ns | >0.9999 |
| 3DP-BRU vs. 3DP-OCP | ns | >0.9999 |
| **Effect size** (Epsilon squared, ε²) = 0.82 | | |

*Data were analyzed using one-way ANOVA followed by Bonferroni multiple comparisons test.*

**S23** **Table** Quantitative number of TRAP positive cells at 12 weeks

| **Group** | **Mean** | **SEM** | **n** |
| --- | --- | --- | --- |
| 3DP-HA | 23.00 | 2.21 | 9 |
| BBG | 4.78 | 1.57 | 9 |
| FDBA | 2.59 | 1.80 | 9 |
| 3DP-MO | 9.41 | 1.03 | 9 |
| 3DP-BRU | 10.30 | 2.06 | 9 |
| 3DP-OCP | 16.67 | 1.99 | 9 |

*Data are presented as mean ± SEM (n = 9 per group). Statistical analysis was performed using one-way ANOVA followed by Bonferroni multiple comparisons test.*

**S24** **Table** Statistical comparisons of quantitative number of TRAP positive cells at 12 weeks

| **Comparison** | **Summary** | **Adjusted p Value** |
| --- | --- | --- |
| 3DP-HA vs. 3DP-MO | ns | 0.0951 |
| 3DP-HA vs. 3DP-BRU | ns | 0.1484 |
| 3DP-HA vs. 3DP-OCP | ns | >0.9999 |
| BBG vs. 3DP-MO | ns | >0.9999 |
| BBG vs. 3DP-BRU | ns | >0.9999 |
| BBG vs. 3DP-OCP | * | 0.0233 |
| FDBA vs. 3DP-MO | ns | 0.4620 |
| FDBA vs. 3DP-BRU | ns | 0.3137 |
| FDBA vs. 3DP-OCP | ** | 0.0013 |
| 3DP-MO vs. 3DP-BRU | ns | >0.9999 |
| 3DP-MO vs. 3DP-OCP | ns | >0.9999 |
| 3DP-BRU vs. 3DP-OCP | ns | >0.9999 |
| **Effect size** (Epsilon squared, ε²) = 0.62 | | |

*Data were analyzed using one-way ANOVA followed by Bonferroni multiple comparisons test.*
