# Supplementary material for: In vivo evaluation of binder jet 3D-Printed monetite, brushite, and octacalcium phosphate: A comparative study for bone regeneration in a rat calvarial defect model
Source: PLoS One. 2026 May 15;21(5):e0349259. doi: 10.1371/journal.pone.0349259 (PMC13178867; doi:10.1371/journal.pone.0349259)
Supplement: S21 Table — (DOCX) [file pone.0349259.s021.docx]

**S21 Table Quantitative number of TRAP positive cells at 4 weeks**

| **Group** | **Mean (cells/HPF)** | **SEM** | **n** |
| --- | --- | --- | --- |
| 3DP-HA | 59.89 | 8.16 | 9 |
| BBG | 2.89 | 1.01 | 9 |
| FDBA | 2.19 | 1.50 | 9 |
| 3DP-MO | 37.33 | 2.73 | 8 |
| 3DP-BRU | 18.07 | 2.61 | 9 |
| 3DP-OCP | 31.67 | 2.39 | 9 |

*Data are presented as mean ± SEM (n =8- 9 per group). Statistical analysis was performed using one-way ANOVA followed by Bonferroni multiple comparisons test.*

**One sample from the 3DP-MO group was excluded due to tissue processing artifacts.*
