# Supplementary material for: In vivo evaluation of binder jet 3D-Printed monetite, brushite, and octacalcium phosphate: A comparative study for bone regeneration in a rat calvarial defect model
Source: PLoS One. 2026 May 15;21(5):e0349259. doi: 10.1371/journal.pone.0349259 (PMC13178867; doi:10.1371/journal.pone.0349259)
Supplement: S23 Table — (DOCX) [file pone.0349259.s023.docx]

**S23 Table Quantitative number of TRAP positive cells at 12 weeks**

| **Group** | **Mean (cells/HPF)** | **SEM** | **n** |
| --- | --- | --- | --- |
| 3DP-HA | 23.00 | 2.21 | 9 |
| BBG | 4.78 | 1.57 | 9 |
| FDBA | 2.59 | 1.80 | 9 |
| 3DP-MO | 9.41 | 1.03 | 9 |
| 3DP-BRU | 10.30 | 2.06 | 9 |
| 3DP-OCP | 16.67 | 1.99 | 9 |

*Data are presented as mean ± SEM (n = 9 per group). Statistical analysis was performed using one-way ANOVA followed by Bonferroni multiple comparisons test.*
