# Supplementary material for: In vivo evaluation of binder jet 3D-Printed monetite, brushite, and octacalcium phosphate: A comparative study for bone regeneration in a rat calvarial defect model
Source: PLoS One. 2026 May 15;21(5):e0349259. doi: 10.1371/journal.pone.0349259 (PMC13178867; doi:10.1371/journal.pone.0349259)
Supplement: S24 Table — (DOCX) [file pone.0349259.s024.docx]

**S24 Table Statistical comparisons of quantitative number of TRAP positive cells at 12 weeks**

| **Comparison** | **Summary** | **Adjusted p Value** |
| --- | --- | --- |
| 3DP-HA vs. 3DP-MO | ns | 0.0951 |
| 3DP-HA vs. 3DP-BRU | ns | 0.1484 |
| 3DP-HA vs. 3DP-OCP | ns | >0.9999 |
| BBG vs. 3DP-MO | ns | >0.9999 |
| BBG vs. 3DP-BRU | ns | >0.9999 |
| BBG vs. 3DP-OCP | * | 0.0233 |
| FDBA vs. 3DP-MO | ns | 0.4620 |
| FDBA vs. 3DP-BRU | ns | 0.3137 |
| FDBA vs. 3DP-OCP | ** | 0.0013 |
| 3DP-MO vs. 3DP-BRU | ns | >0.9999 |
| 3DP-MO vs. 3DP-OCP | ns | >0.9999 |
| 3DP-BRU vs. 3DP-OCP | ns | >0.9999 |
| **Effect size** (Epsilon squared, ε²) = 0.62 | | |

*Data were analyzed using one-way ANOVA followed by Bonferroni multiple comparisons test.*
